# Losartan Improves Memory, Neurogenesis and Cell Motility in Transgenic Alzheimer’s Mice

**DOI:** 10.3390/ph14020166

**Published:** 2021-02-20

**Authors:** Henning Johannes Drews, Roman Klein, Ali Lourhmati, Marine Buadze, Elke Schaeffeler, Thomas Lang, Torgom Seferyan, Leah R. Hanson, William H. Frey II, Tom C.G.M. de Vries, Inge A.E.W. Thijssen-van Loosdregt, Christoph H. Gleiter, Matthias Schwab, Lusine Danielyan

**Affiliations:** 1 Department of Clinical Pharmacology, University Hospital of Tuebingen, 72076 Tuebingen, Germany; drews.henning@gmail.com (H.J.D.); roman-klein@web.de (R.K.); alilourhmati@yahoo.de (A.L.); buadze@hotmail.com (M.B.); Christoph.Gleiter@med.uni-tuebingen.de (C.H.G.); Matthias.Schwab@ikp-stuttgart.de (M.S.); 2 Dr. Margarete Fischer-Bosch-Institute of Clinical Pharmacology, 70376 Stuttgart, Germany; Elke.Schaeffeler@ikp-stuttgart.de (E.S.); Dr.Thomas.Lang@gmx.de (T.L.); 3 H. Buniatian Institute of Biochemistry, National Academy of Sciences of the Republic of Armenia (NASRA), Yerevan 0019, Armenia; seferyant@yahoo.com; 4 HealthPartners Center for Memory and Aging, HealthPartners Neurosciences, St. Paul, MN 55130, USA; Leah.R.Hanson@healthpartners.com (L.R.H.); alzheimr@umn.edu (W.H.F.II); 5 CytoSMART, 5611 AZ Eindhoven, The Netherlands; tom.devries@cytosmart.com (T.C.G.M.d.V.); inge.thijssen@cytosmart.com (I.A.E.W.T.-v.L.); 6 Department of Biochemistry and Neuroscience Laboratory, Yerevan State Medical University, Yerevan 0025, Armenia; 7 Department of Biochemistry and Pharmacy, University of Tuebingen, 72074 Tuebingen, Germany; 8 Department of Clinical Pharmacology, Yerevan State Medical University, Yerevan 0025, Armenia

**Keywords:** angiotensin receptor, amyloid beta, neuroinflammation, cell migration, cell motility, neprilysin, BACE1, 3xTg-AD, APP/PS1, cholinergic

## Abstract

Angiotensin receptor blockers (ARBs) have demonstrated multiple neuroprotective benefits in Alzheimer’s disease (AD) models. However, their beneficial effects on memory deficits, cholinergic activity, neurogenesis and Amyloid beta (Aβ) clearance reveal significant interstudy variability. The delivery route can impact not only delivery but also targeting and therapeutic efficacy of ARBs. Our previous findings on the beneficial effects of intranasally delivered losartan in the APP/PS1 model of AD prompted us to explore the influence of the delivery route by employing here the systemic administration of losartan. Consistent with our previous results with intranasal losartan, repeated intraperitoneal administration (10 mg/kg) resulted in a remarkable decrease in Aβ plaques and soluble Aβ42, as well as inflammatory cytokines (IL-2, IL-6 and TNFα). The Aβ reduction can be ascribed to its facilitated degradation by neprilysin and diminished generation by BACE1. Losartan increased neurogenesis in vivo and in vitro and improved migratory properties of astrocytes isolated from adult transgenic AD mice. In summary, this data together with our previous results suggest therapeutic features of losartan which are independent of delivery route. The improvement of cell motility of Aβ-affected astrocytes by losartan deserves further *in vivo* investigation, which may lead to new strategies for AD treatment.

## 1. Introduction

The local renin-angiotensin system (RAS) in the CNS has been proposed to play a critical role in the pathological hallmarks of Alzheimer’s disease (AD), such as memory deficits, neuroinflammation and impaired neurogenesis. While the stimulation of angiotensin receptor 1 (AT1R) has been suggested to cause cognitive impairment, neuronal cell death and neuroinflammation (for review see [1,2]), the AT1R blockers (ARBs) were shown to blunt these effects when administered systemically or intranasally in rodent models of AD [3,4,5,6]. Despite the uniform confirmation of AT1R blockade (ARB)-mediated memory improvement and anti-inflammatory effects in AD models, the impact of this drug class on amyloid (Aβ) plaque pathology remains controversial. Studies using intranasal delivery of ARBs in transgenic mice carrying both human amyloid precursor protein (APP) and presenilin 1 (PS1) mutations (5XFAD and APP/PS1 mouse strains), as well as in spontaneously hypertensive rats, suggested an ARB-mediated decrease in soluble Aβ [7] and plaque area [3,4,8], whereas others employing mice with the Swedish APP mutation and systemic drug delivery reported no influence of ARBs on the brain content of Aβ [5,9]. In addition to reduced Aβ plaque area, our previous study demonstrated a decrease in the plasma level of inflammatory cytokines after two months of intranasal treatment with losartan in APP/PS1 mice [3]. However, the question of whether losartan at a dose lacking blood pressure (BP)-lowering activity can improve memory deficits and local inflammation in the brain remained unanswered. Since memory improvement has been previously shown in transgenic AD mice following treatment with telmisartan [4], the potential effect of losartan on memory deficits in APP/PS1 mice reported here would support the notion of a class-wide effect of sartans on cognitive performance in transgenic mouse models of AD.

Furthermore, to close the gaps in our understanding of the mechanisms involved in Aβ-lowering effects of ARBs in AD, we sought to investigate the influence of losartan on the key enzymatic components of Aβ generation (beta secretase 1, BACE1) and degradation (neprilysin, NEP).

Brain AD pathology is strongly associated with changes in neurotransmitter levels such as acetylcholine (ACh) and glutamate (Glu) in both AD patients and in rodent AD models [10,11,12,13]. To find out whether the memory improving feature of ARBs is associated with their modulating effects on the level of Glu and ACh, we assessed the levels of the key enzyme in ACh degradation and glutamine synthetase (GS), the enzyme that utilizes Glu to form glutamine.

The present findings provide insights into the neuroprotective and memory improving features of losartan. Our data demonstrate that losartan is capable of targeting proteins interconnecting the cholinergic activity, Glu excitotoxicity, neurogenesis and inflammation. Furthermore, the previously demonstrated decrease in Aβ by losartan has been shown to involve mechanistically the key enzymes of Aβ generation and degradation, BACE1 and neprilysin.

## 2. Results

### 2.1. ARB Improves the Spatial Memory Deficit in APP/PS 1 Mice Independent of Its BP-Lowering Effect

**Figure 1:**
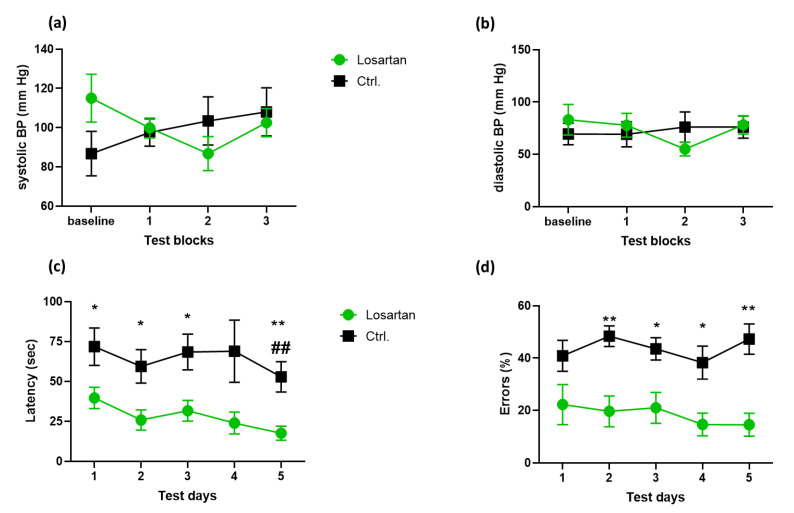
Blood pressure (BP) and cognitive performance of losartan and vehicle treated APP/PS1 mice. Seven-month-old APP/PS1 mice (*n* = 9 in losartan and *n* = 8 in control group) were assessed for blood pressure and spatial working memory in losartan and vehicle treated (Ctrl.) groups. (**a**,**b**) systolic and diastolic blood pressure measured prior (baseline) and during drug (vehicle treatment (test blocks 1–3); (**c**) Latency to reach the goal arm and (**d**) number of incorrect choices in forced choice alternation T-maze assessed at the last 5 days of losartan or vehicle treatment. (**c**,**d**) Two-way ANOVA with Holm Sidak’s multiple comparisons test *p* < 0.05 (*), *p* < 0.01 (**) comparison between losartan and Ctrl. group; (##) comparison between day 1 and day 5 within the losartan-treated group.

To prove that the observed effects of losartan on the memory, protein and gene expression are independent of its blood pressure (BP)-lowering feature, we measured BP at the beginning (baseline in Figure 1a,b) and during the period of losartan administration (blocks 1–3 in Figure 1a,b). Both systolic (Figure 1a) and diastolic (Figure 1b) values remained nearly equal to the vehicle controls over the entire period of losartan administration.

Spatial working memory was tested in the last week of drug treatment (days 26 to 30 shown as days 1–5 of T-maze testing in Figure 1 c,d) by forced choice alternation T-maze test. This test has been previously reported to be highly sensitive to hippocampal dysfunction [14,15]. A significant improvement in cognitive performance reflected by decreased latency to choose the correct arm (Figure 1c) and the number of incorrect arm choices (Figure 1d) was seen in the losartan treated group in comparison to vehicle treated controls. The highest effect was observed on the last day (day 30) of testing, where losartan improved not only the latency and error numbers in comparison to the values of the same day for the vehicle group (cf. Ctrl. vs. losartan group at day 5 in Figure 1c,d), but also to the performance within the drug-treated group when the first (day 26) and the last day of the T-maze test were compared (cf. losartan group on day 1 vs. day 5 in Figure 1c,d).

### 2.2. Anti-Inflammatory Effects of ARB in APP/PS1 Mice

**Figure 2:**
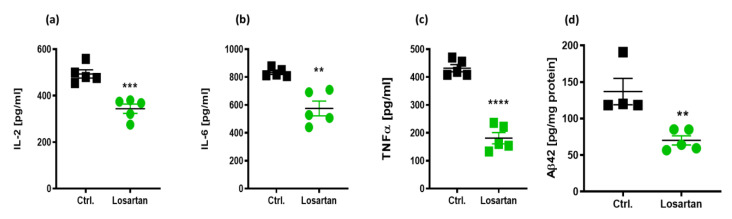
Cerebral content of inflammatory cytokines and soluble beta Amyloid peptide 42 (Aβ42) in losartan and vehicle treated APP/PS1 mice. Brain homogenates of APP/PS1 mice (*n* = 5) were assessed for the levels of inflammatory cytokines. IL-2 (**a**), IL-6 (**b**), and TNFα (**c**) in losartan and vehicle treated (Ctrl.) groups. (**d**) Brain content of soluble Aβ42 in losartan and vehicle treated APP/PS1 mice. *t*-test, ** p < 0.01, *** *p* < 0.0005, **** *p* < 0.0001.

Multiplex analysis of interleukin-a, IL-1b, IL-2, IL-4, IL-5, IL-6, IL-10, IL-12, tumor necrosis factor α (TNFα), interferon γ (IFNγ), and granulocyte-macrophage colony stimulating factor (GM-CSF) in the brain homogenates of APP/PS1 mice revealed that only IL-2, IL-6 and TNFα were significantly decreased by losartan treatment (Figure 2a–c), while the remining factors (data not shown) were below the detection range in the used test in both treatment groups.

The brain content of Aβ42 in APP/PS1 mice, which is known to be elevated in comparison to their wild type controls [16], was also prominently decreased by losartan (Figure 2d).

### 2.3. ARB Increases Neurogenesis Markers and EPO Production in APP/PS1 Mice

**Figure 3:**
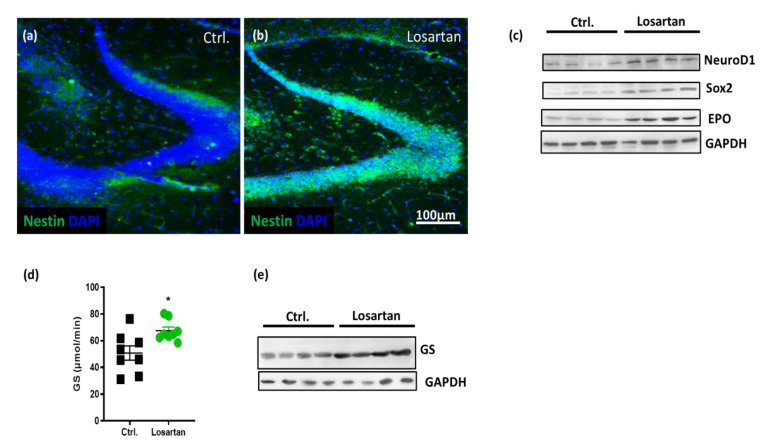
Losartan-mediated changes in neurogenesis and neuroprotection markers in APP/PS1 mice. (**a**,**b**) Expression of nestin (green) in the hilus and granular cell layer of the denate gyrus in brain sections of losartan vs. vehicle (Ctrl.) treated APP/PS1 mice. Cell nuclei are stained with 4´,6 diamidino-2-phenylindole (DAPI, blue), scale bar shown in (**b**) corresponds to the images shown in (**a**) and (**b**). (**c**) Cerebral content of Neuronal Differentiation 1 protein (NeuroD1), SRY-related HMG-box 2 (Sox2) and erythropoietin (EPO) assessed in brain homogenates of APP/PS1 mice (*n* = 4) by Western Blot. (**d**,**e**) Enzymatic activity (*n* = 8) and expression level of glutamine synthetase (GS) in brain homogenates of losartan and vehicle (Ctrl.) treated APP/PS1 mice (*n* = 4). Statistical analysis in (**d**): *t*-test, * *p* < 0.05.

Our previous results and the results from other groups provided evidence for neurogenesis supportive effects of ARBs in different models of CNS diseases, including AD [5,7,17]. Here, we examined the neurogenesis biomarkers nestin, SRY-related HMG-box 2 (Sox-2) and Neuronal Differentiation 1 protein (NeuroD1). A remarkable increase in the expression of nestin was observed in the hilus and granular cell layer of the dentate gyrus in the losartan treated group as assessed by immunohistochemistry (Figure 3a,b). Western Blots from total brain homogenates demonstrated losartan induced increases in the expression of Sox 2 and NeuroD1 (Figure 3c). Acknowledging the neuroprotective effects of erythropoietin (EPO) on the survival and regeneration of neurons in AD [18,19] and the previously demonstrated reduction in EPO/EPO receptor expression in the brain of APP/PS1 mice [20], we assessed its cerebral level in vehicle vs. losartan treated APP/PS1 mice. The APP/PS1 mice responded to losartan with an upregulation of EPO (Figure 3c).

### 2.4. ARB Improves the Glutamate-Metabolizing Function in the CNS of APP/PS1 Mice

Aβ-induced release of glutamate (Glu) from astrocytes [21] is suggested to contribute to excitotoxic effects of Glu in AD [21,22]. Moreover, a downregulation of GS expression in hippocampal astrocytes in transgenic mice AD is proposed to be critically involved in the disruption of Glu homeostasis in AD, and by influencing Glu transmission to contribute to cognitive deficits [22,23]. The fact that losartan increases the expression of EPO in the brains of APP/PS1 mice shown here as well as our previous data demonstrating the critical role of EPO in the regulation of both the amount and activity of GS in aged astrocytes [20] hint at the possible modulation of GS expression by ARB via its effect on the intracerebral content of EPO. Indeed, the losartan treated group displayed both an enhanced activity and level of GS in brain homogenates of APP/PS1 mice (Figure 3d,e).

### 2.5. ARB Regulates the Expression of BACE1, AChE, ChAT and Neprilysin in the Brains of APP/PS1 Mice

**Figure 4:**
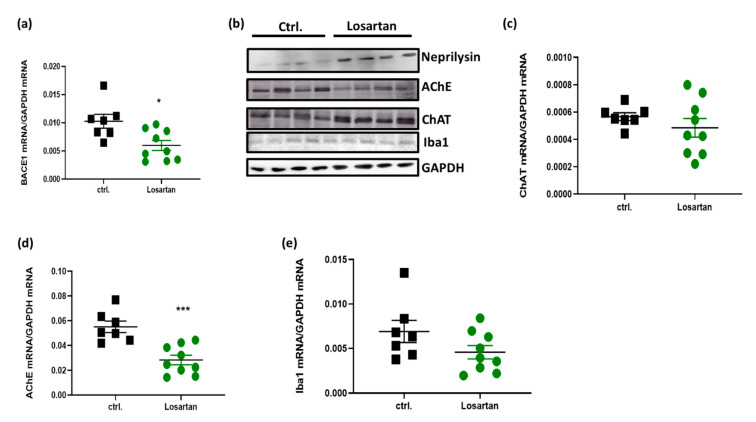
Expression of factors regulating cholinergic activity, Aβ clearance and microglial function in losartan-treated APP/PS1 mice. (**a**) qPCR of β secretase 1 (BACE1) mRNA (*n* = 7 in control and *n* = 9 in losartan treated group); (**b**) Western blot of neprilysin, Acetylcholine esterase (AChE), Choline acetyltransferase (ChAT) and ionized calcium-binding adapter molecule 1 (Iba1), *n* = 4; (**c**–**e**) qPCR of AChE, ChAT and Iba1 mRNA (*n* = 7 in control and *n* = 9 in losartan treated group). Statistical analysis in (**a**) and (**c**–**e**): *t*-test, * *p* < 0.05, *** *p* < 0.0005.

Since losartan was capable of decreasing the soluble Aβ42 fragment in APP/PS1 mice, we tested whether this effect can be ascribed to changes in the generation of amyloidogenic species by Aβ generating enzymes such as BACE1 or to the facilitated degeneration of Aβ by a key degrading enzyme, neprilysin. qPCR data and Western Blot analyses revealed that BACE1 mRNA was reduced (Figure 4a), while neprilysin (Figure 4b) protein was enhanced following the administration of losartan.

In a view of the improved cognitive performance of APP/PS1 mice treated with losartan (Figure 1c,d), we sought to examine whether this effect is dependent on losartan-mediated changes in cholinergic activity. ACh generating enzyme ChAT was upregulated as shown by Western Blot (Figure 4b), while the content of ChAT mRNA remained unchanged in the losartan-treated group (Figure 4c). Concomitantly, losartan was capable of reducing the mRNA of ACh-degrading enzyme AChE (Figure 4d).

The decrease in anti-inflammatory cytokines shown here led us to test whether these losartan-evoked changes can affect the motility and phagocytotic function of microglia, which are both impaired in mice with AD-like pathology [24]. Here, we used ionized calcium-binding adapter molecule (Iba1) as a marker of microglial functionality. Iba1 is known not only as a common marker of microglia since it also plays a crucial role in organizing actin filaments into networks and in building structures essential for microglial migration and phagocytosis, such as lamellipodia, filopodia, and membrane ruffles [25,26]. As shown in Figure 4, Iba-1 expression remained unchanged in the losartan treated group in comparison to the control at both the mRNA and protein levels (Figure 4b,e).

**Figure 5:**
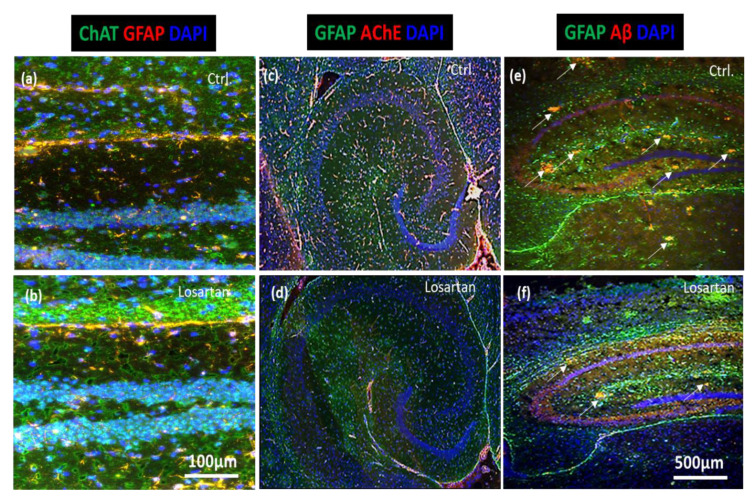
Losartan-mediated changes in neurogenesis and neuroprotection markers in the hippocampus of APP/PS1 mice. (**a**,**b**) Immunofluorescent analysis of ChAT (green) and glial fibrillary acidic protein (GFAP) (red) in losartan vs. vehicle (Ctrl.) treated APP/PS1 mice. (**c**,**d**) Expression of AChE (red) and GFAP (green) in losartan/vehicle treated APP/PS1 mice. (**e**,**f**) Aβ plaques (red) and GFAP staining of losartan and vehicle (Ctrl.) treated APP/PS1 mouse brain sections. Cell nuclei are stained with DAPI (blue), scale bar in (**b**) corresponds to the images shown in (**a**) and (**b**), scale bar in (**f**) corresponds to (**c**–**f**). (**a**–**f**) Representative images out of 10 sections per brain taken from *n* = 3 mice analyzed per treatment group.

Consistent with the results from qPCR and Western Blots of cholinergic markers, as well as the assessment of Aβ42 by ELISA, immunofluorescent analyses of brain sections demonstrated a prominent increase in ChAT expression (Figure 5a,b), whereas AChE and Aβ plaques were markedly reduced in losartan treated animals in comparison to the vehicle treated controls (Figure 5c vs. Figure 5d and Figure 5e vs. Figure 5f, respectively). 

Altogether, our in vivo results indicate that AT1-R blockade favors increased cholinergic activity and decreased inflammatory milieu in AD-affected brain tissue, which in turn could explain the improved cognitive performance of losartan treated mice. In addition, losartan decreased soluble Aβ and plaque number, most likely as a result of facilitated degradation by neprilysin and slowed generation via BACE1.

### 2.6. ARB Enhances the Production of ACh, Neurogenesis and Cell Motility In Vitro

To investigate the direct effects of ARB on brain cells, astroglial primary cultures (APC) containing astrocytes, neural precursors and neurons were isolated from wild type (WT) mice and from triple transgenic AD mice (3xTg-AD).

**Figure 6:**
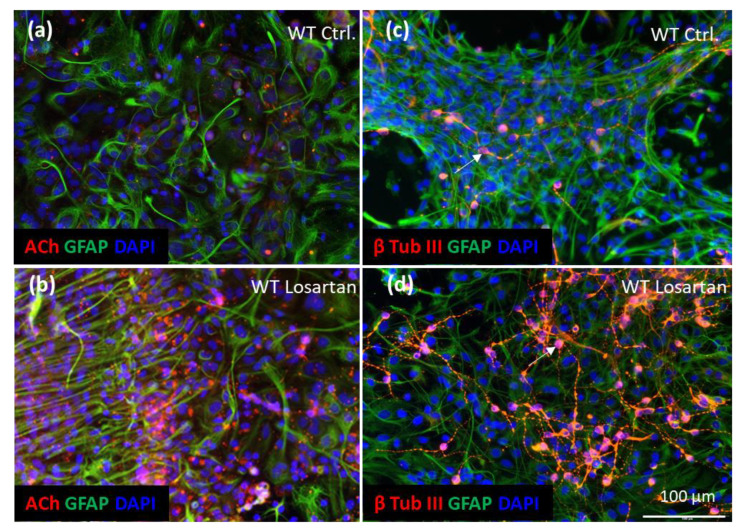
Losartan-induced neurogenesis and ACh production in wild type (WT) astroglial primary cultures (APC). (**a**,**b**) Immunofluorescent analysis of GFAP (green) and ACh (red) in losartan vs. vehicle (Ctrl.) treated WT APC. (**c**,**d**) Expression of β tubulin III (red) and GFAP (green) in losartan/vehicle treated WT APC. Arrow in (**d**) indicates young β tubulin III positive neuron in losartan treated culture. Cell nuclei are stained with DAPI (blue), scale bar in (**d**) corresponds to the images shown in (**a**–**d**). (**a**–**d**) Representative images out of *n* = 4 cover slips stained with ACh/GFAP or β tubulin III/GFAP in each treatment condition.

WT APC responded to the administration of 1 μM losartan with a prominent increase in the number of ACh-positive cells, the majority of which were glial fibrillary acidic protein (GFAP) negative (Figure 6a vs. Figure 6b).

Since besides astrocytes WT APC also contain neural progenitors and young neurons, the only reasonable explanation for the enhanced population of ACh-positive cells, other than astrocytes, was that losartan induced the proliferation and differentiation of neural precursors leading to an increase in the number of young neurons. This was demonstrated by immunofluorescent staining of APC with the marker of young neurons, β Tubulin III. Indeed, a dramatic increase in the number of β Tubulin III positive neurons was identified in the losartan treated APC, while only a small number of young neurons could be seen in control cultures (Figure 6c vs. Figure 6d).

Similar to our in vivo data, losartan-treated 3xTg-AD APC displayed an enhanced expression of ChAT and reduced levels of BACE 1 shown by Western Blot (Figure 6a) concomitant to the increased number and intensity of neprilysin expression assessed by immunofluorescence (Figure 6b–d).

**Figure 7:**
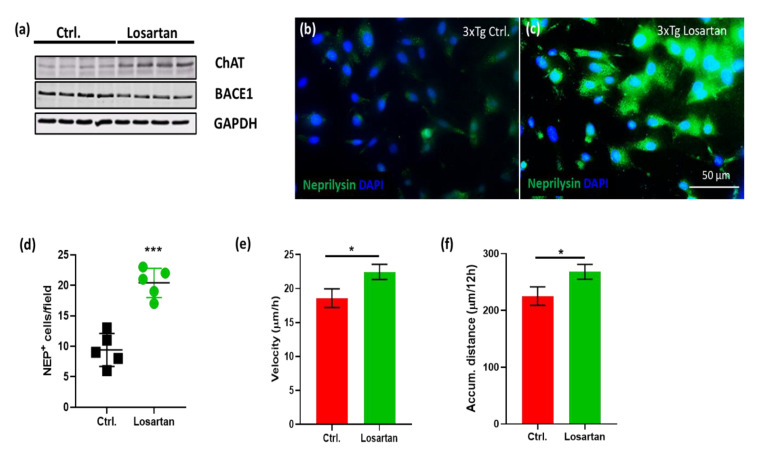
In vitro effects of losartan on triple transgenic Alzheimer’s disease (AD) mice (3xTg-AD) APC. (**a**,**b**) Western Blot of ChAT and BACE1 in losartan vs. vehicle (Ctrl.) treated 3xTg-APC. (**b**,**c**) Expression of neprilysin (green) in losartan (3xTg losartan) and vehicle (3xTg Ctrl.) treated 3xTg-APC. Cell nuclei are stained with DAPI (blue), scale bar in (**d**) corresponds to the images shown in (**b**,**c**). (**d**) Quantification of neprilysin positive cells in 3xTg-APC (*n* = 5). (**e**) Velocity of migrated cells in the control (ctrl.) and losartan treated 3xTg-APC. From the cells shown in videos in Supplementary Figure S1, we identified and analyzed 32 cells per treatment group (*n* = 32) which were trackable for at least 12h; (**f**) accumulated distance calculated for cells described in (**e**); Statistical analysis in (**d**–**f**): *t*-test, * *p* < 0.05, *** *p* < 0.0005.

Astrocytes are potent metabolizers of Aβ [27]. Assuming that Aβ plaque targeting activity may be dependent on their migratory features, we investigated whether losartan may affect astrocyte motility and migration velocity. Live imaging of 3xTg-APC over a period of 24 h (Supplementary Figure S1) revealed that the velocity and the motility (reflected by accumulated distance) of losartan treated cells were greater than that of controls (Figure 7e,f). 

## 3. Discussion

This study elucidated multifaceted neuroprotective features of systemically administered losartan in a transgenic model of AD as demonstrated by: (1) improved spatial working memory; (2) increased neurogenesis; (3) enhanced cholinergic activity; (4) reduced neuroinflammation; (5) enhanced Glu- and Aβ-clearance and (6) the improvement of Aβ-affected astroglial migration. Further, our data show that losartan modulates the factors (such as EPO) interconnecting some of the aforementioned processes (neurogenesis, Glu-clearance and neuroinflammation), as well as the proliferation and migrational capacity of astrocytes affected by Aβ.

Here, we show that similar to telmisartan’s effect in the 5-familial AD mice (5XFAD) mouse line [8], intraperitoneally applied losartan is capable of improving working memory in APP/PS1 mice. Notably, also oral administration of losartan in a dose lacking BP-lowering effect has also been reported to improve spatial and learning memory deficits in APP mice [5]. As for the majority of ARBs, the blood-brain-barrier (BBB) permeability of losartan is poor [28]. However, according to our data and previous reports on the neuroprotective effects of losartan, telmisartan and candesartan [3,6,8,29], it can be concluded that the concentration of ARBs delivered to the brain after systemic or intranasal administration is enough to trigger the effects described here and in the literature. Moreover, 10 mg/kg of systemically administered losartan appears to inhibit 60–80% of radiolabeled angiotensin II binding to AT receptors in different brain areas of rats 24 h after losartan administration [30].

Our previously published results on the Aβ-lowering benefit of intranasally administered losartan [3] in the same experimental setting as used here (aside from the route of administration of losartan) are also in line with those reported for telmisartan and candesartan in 5XFAD mice [8,29]. The present data, however, show that GM-CSF, IL1β, IL-10 and IL-12 were below the detection range in the brain homogenates of APP/PS1 mice, while serum levels of GM-CSF, IL1β, IL-10 and IL-12 were affected by AD pathology and modulated by intranasally administered losartan [3]. Oppositely, the levels of IL-6 and TNFα were decreased by losartan in the brains of APP/PS1 mice as shown here, but could not be detected in the serum of either the intranasal losartan treated group or in the controls examined in our previous study [3]. This suggests that there is no correlation between the blood and brain content of inflammatory cytokines as well as in losartan’s effects on them, at least in the experimental setting reported in the present paper and in our previous study on intranasally administered losartan [3].

The facilitated degradation of Aβ by neprilysin proposed here could not be observed in 5XFAD mice treated with candesartan, since the level of neprilysin remained unchanged in the drug-treated group [29]. Notably, in spontaneously hypertensive stroke prone rats (SHRSP), a model that does not exert Aβ plaque pathology but is associated with an increase in soluble Aβ in the brain, intranasal administration of ARBs has been reported to upregulate neprilysin along with reducing the brain levels of soluble Aβ [7]. In contrast to this observation, in rats with aluminum-induced AD-like pathological changes, the systemic administration of telmisartan did not lead to changed neprilysin levels, while showing the improvement of memory and a decrease in the cerebral content of Aβ42 [6]. The inconsistencies in reported ARB effects on the brain content of Aβ and neprilysin may possibly be explained by variability in the extent of Aβ pathology across different transgenic and chemically induced AD-like models at different ages, as well as different delivery routes used (systemic vs. intranasal). In this context, ARBs with less lipophilic properties, including losartan [31], are likely to benefit from the blood–brain barrier bypassing routes, such as intranasal delivery. However, as shown here, the systemic administration of losartan appears to be sufficient in terms of delivering losartan to the CNS, reflected by protective effects assessed across different pathologic characteristics of AD, such as neuroinflammation, decreased neurogenesis and cholinergic activity as well as Aβ pathology and Glu-metabolizing function.

To the best of our knowledge, this is the first attempt to demonstrate that the Aβ-decreasing effect of ARBs is likely to involve not only its degrading function via neprilysin, but also the generation of amyloidogenic peptide fragments via BACE1. Moreover, the increase in neprilysin and the enhanced motility of astrocytes following losartan treatment indicate an improvement of their well-known feature to protect neurons against Aβ toxicity [32]. The decreases in BACE1 mRNA and ChAT protein in losartan treated APP/PS1 are concordant with the BACE1 and ChAT protein content of losartan treated astrocytes isolated from 3xTg-AD mice. The reason why losartan induced an increase in ChAT solely at the protein level remains to be elucidated in the future. It is unlikely to be due to the decreased degradation of ChAT via ubiquitination-proteolytic degradation [33], since this process appears to be impaired rather than increased in AD [34]. Notably, the interconnection of losartan-induced cholinergic activity (shown by AChe decrease) with downregulated BACE1 complies with the previously suggested regulation of BACE1 content by muscarinic receptor 1 in the brain of triple transgenic AD mice [35]. Similar to the results in APP/PS1 mice, a significant increase in ChAT by losartan has been demonstrated in the SHRSP model at the mRNA and protein level [7]. This agrees with another report on the impact of systemically administered losartan and telmisartan on cholinergic activity in scopolamine-treated rats with memory impairment [36]. Taken together, our data along with previous reports suggest a role for the local RAS in the brain, not only in the inhibition of cholinergic activity via an Angiotensin II-mediated decrease in the ACh release [1], but also the stimulatory effect through the AT1R-mediated modulation of ACh generation (by ChAT) and degradation (by AChE).

Cholinergic activation has been previously proven to promote the proliferation of hippocampal neural stem cells (NSC) [37]. Thus, the upregulation of neurogenesis markers Sox2 and NeuroD1 in losartan treated APP/PS1 mice, as well as an increase in the number of young neurons/neuronal progenitors in losartan treated brain primary cultures demonstrated here, provides another mechanistic link between the local RAS, neurogenesis and cholinergic system in the brain, where the AT1R acts as a negative regulator of the ACh level, and its blockade consequently enhances neurogenesis in degenerated brain.

**Figure 8:**
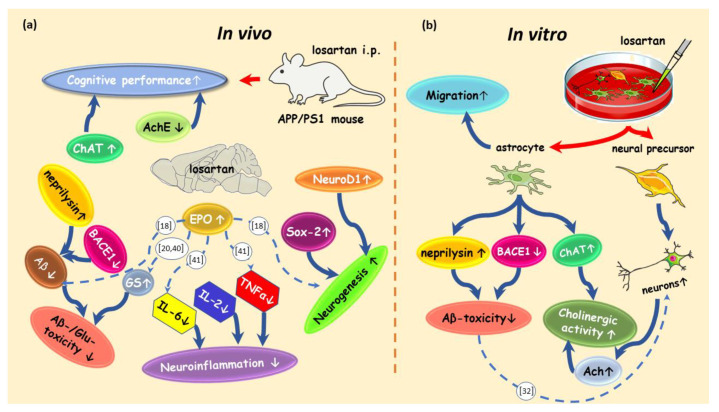
Schematic illustration of processes influenced by losartan in an AD-like pathology in vivo and in vitro. (**a**) In vivo effects of losartan in a transgenic model of AD (APP/PS1 mouse). Intraperitoneal administration of losartan led to a decrease in: Acetylcholine esterase (AchE), TNFα, IL-6, IL-2, β secretase 1 (BACE1), and beta amyloid (Aβ), and to an increase in Choline acetyltransferase (ChAT), glutamine synthetase (GS), neprilysin, SRY-related HMG-box 2 (Sox-2), Neuronal Differentiation 1 protein (NeuroD1) and erythropoietin (EPO). Blue solid arrows connect the processes (neurogenesis, neuroinflammation, cholinergic activity and Aβ/Glutamate (Glu)-toxicity) with their respective markers. Dashed blue arrows show the effect of EPO on GS, TNFα, IL-6 and neurogenesis evidenced by the literature indicated in white circles; (**b**) in vitro effects of losartan on the astrocytes and neural precursors. Losartan induced the differentiation of neurons from neural precursors, increased the Acetylcholine (ACh) in neurons, decreased the expression of BACE1 and upregulated ChAT and neprilysin in astrocytes. Losartan improved the motility and migration of astrocytes. This schematic drawing was created using art elements from Servier Medical Art Commons Attribution 3.0 Unported License. Servier Medical Art by Servier is licensed under a Creative Commons Attribution 3.0 Unported License.

Acknowledging the prominent and multifactorial role of Glu in the pathogenesis of AD, we found that blocking AT1R in APP/PS1 mice by losartan enhances the amount and activity of GS, which plays a pivotal role in the homeostasis of Glu in the brain [38]. In the present study, the factor interconnecting the Glu clearance, neurogenesis and neuroinflammation, EPO, has been shown to be upregulated in the CNS of losartan treated animals. EPO is known to decrease inflammatory cytokines and to enhance the activity and expression of GS [18,20,39], as well as to increase neurogenesis in different models of CNS degeneration including AD [18,19,20,40]. Particularly, in an in vivo model of AD, intranasal administration of EPO led to a decrease in TNFα and soluble and insoluble fractions of Aβ [18]. The modulating effects of EPO on the cerebral production of TNFα and IL-6 have been proposed to involve the TLR4–nuclear factor κB (NF-κB) signaling pathway [41]. Of note, IL-6 inhibits neurogenesis [42], which supports the notion that the neurogenesis inducing effect of EPO is likely to involve its IL-6 reducing effect. Together with the literature, our data hint at a link between the local systems in the CNS, EPO receptor system and RAS, where the blockade of ATR1 increases the cerebral content of EPO, which in turn enhances the neurogenesis-inducing, inflammation decreasing and Aβ-lowering effects of an ARB (Figure 8).

The direct effects of losartan on the generation and differentiation of neuronal progenitors, as well as on the content of ACh, have been demonstrated here in vitro using WT and 3xTg-AD brain cultures. Of note, ACh has been previously shown to be decreased in 3xTg-AD mice [43]. In this context, it is also noteworthy that ATR1 blockade appears to reduce the cerebral levels and activity of AChE in rats with deoxycorticosterone acetate (DOCA)-salt induced experimental hypertension [44]. Consistent with our in vitro and in vivo data showing the neurogenic effect of losartan, previous reports demonstrated losartan- and candesartan-enhanced neurogenesis in hypertensive rats [7,17] and valsartan-induced restoration of hippocampal neurogenesis in unpredictable chronic mild stress mice [45], suggesting a drug class-wide effect on neurogenesis across different models of CNS injury.

Astrocytes have been suggested to be potent metabolizers of Aβ [27,46], and thus valuable therapeutic targets for enhancing Aβ-clearance in AD. However, whether a general enhancement of astroglial motility will lead to more effective targeting of Aβ plaques in vivo remains a matter for future investigations. Previous studies have demonstrated the improvement of Aβ plaques and soluble fragments’ clearance from the brains of 3xTg-AD and APP/PS1 mice treated with mesenchymal stem cells (MSC) by simply enhancing the migratory properties of MSC via the selection of highly migratory subpopulations for cell transplantation [47]. ARBs as potential enhancers of cell migration may be tested for their influence on the migratory properties of different cell therapeutics (adult stem cells, induced pluripotent stem cells, etc.) in the near future. As outlined in the schematic drawing summarizing in vivo and in vitro evidence for losartan’s multiple protective features in AD (Figure 8), further studies are needed to fully explore ARBs’ therapeutic potency and the mechanisms involved in their capacity to slow AD progression. Considering the literature evidence for a strong influence of Aβ burden on the functionality of different types of neural cells, future investigations of losartan on these functions affected by Aβ pathology will help to not only explore the mechanisms behind ARB-provided neuroprotection, but also to estimate their efficacy at different stages of AD. Of such processes affected by Aβ, the hyperactivity of cortical neurons [48], the activation and Aβ-metabolizing function of microglia [49], and the barrier-maintaining features of endothelial cells leading to the changes in BBB permeability [50] would be of special interest.

## 4. Materials and Methods

### 4.1. Animals

The study was performed using double transgenic APP/PS1(APPswe/PS1dE9) male mice obtained from Jackson Laboratories (Bar Harbor, ME). Animals were housed individually with controlled temperature and an artificial light/dark cycle. Food and water were available ad libitum. All animal experiments were performed in accordance with the guidelines of the University of Tübingen for the experimental use of animals and German law for the protection of animals. All experiments were approved by the local Animal Protection Committee, University of Tübingen (approval code: PH1/06). Seven month-old mice were divided into losartan- (*n* = 9) and vehicle (0.9% NaCl)-treated (*n* = 8) APP/PS1 groups. Blood pressure was measured one week before starting the treatment (baseline in Figure 1a,b) and during the last 3 weeks of losartan administration (test blocks 1–3 in Figure 1a,b) by the tail cuff method using a blood pressure analyzer (Hugo Sachs, March-Hugstetten, Germany), as described elsewhere [7]. Losartan (MSD, Haar, Germany) dissolved in 0.9% NaCl was applied intraperitoneally (10 mg/kg body weight) every other day for a period of 1 month. On day 31, the mice were euthanized and the brains were frozen at −80 °C for further processing, as described below.

### 4.2. T Maze Testing

The spatial memory of 7-month-old APP/PS1 mice was assessed during the last 5 days of the treatment period (days 26 to 30 from the beginning of the treatment) by T-maze forced choice alternation after a habituation period of 4 days, in which the mice were allowed to explore the T-maze apparatus for 5 min per day or until the reward (sucrose pellet) placed in the goal arm was found and consumed. The 5-day assessment period was performed in the last week before euthanasia and consisted of 6 paired (“sample” and “choice”) trials per day with a 15 s interval between each paired trial. In the “sample” trial, one of the goal arms was closed and the mouse was forced to select the opposite arm. After 20 s of consuming the reward, the mouse was returned to the start box. The order of the open goal arms in the “sample” trial was counterbalanced. During the choice trial, both goal arms were accessible. The mouse was rewarded only for selecting the previously unvisited arm. The data are presented as the latency to reach the correct arm and the percentage of incorrect choices in the choice trial.

### 4.3. Cell Culture Experiments and Cell Migration Tracking

Astroglial primary cultures (APC) were prepared from brains of newborn wild type (WT) C57/Bl6 mice (Charles River, Sulzfeld, Germany) and 2 month-old 3xTg-AD mice (B6.129-Psen1tm1Mpm Tg(APPSwe,tauP301L) 1Lfa/ Mmjax) obtained from Jackson Laboratory (Bar Harbor, strain provider: Frank LaFerla, University of California Irvine, Irvine, CA, USA). The isolated cell suspension was centrifuged at 300× *g* for 8 min and transferred to 15 mL supplemented cell culture medium. The cells were cultured with Dulbecco’s modified Eagle’s medium supplemented with 10% fetal calf serum, 100 U/mL penicillin, 100 μg/mL streptomycin and 100 mmol/L pyruvate (PAA, Cölbe, Germany). Cultures were maintained under normoxic conditions in a humidified atmosphere containing 10% CO_2_ at 37 °C. On day 13 in vitro, cells were transferred to a 6 cam Petri dishes containing cover slips or to 75 cm² flasks and incubated further for 24 h with or without 1 μM losartan. Thereafter, the cells were either fixated and processed for immunofluorescence analysis or harvested for Western Blotting or tracked for migration, as described below.

The migration of cells was tracked by Lux3 FL microscope (CytoSMART, Eindhoven, Netherlands) placed into an incubator. Prior to starting the video tracking, the medium was refreshed and medium with or without 1μM losartan was added to the culture flasks. The images were taken every 15 min in a total period of 24 h. Velocity and accumulated distance (motility) were determined for the cells that could be tracked for a period of 12 h or longer by the ImageJ plug-in “TrackMate” (Image J, https://imagej.net/TrackMate, accessed on 23 December 2020).

### 4.4. qPCR of APP/PS1 Mouse Brain Samples

High-quality total RNA was isolated from mouse brain tissue using Qiagen RNeasy Isolation Kit (Qiagen). mRNA expression of 4 selected genes (choline acetyltransferase (Chat), acetylcholinesterase (Ache), allograft inflammatory factor 1 (Aif1), the gene coding ionized calcium-binding adapter molecule 1 (Iba1), beta-site APP cleaving enzyme 1 (Bace1)) was quantified in mouse brain tissues using predesigned TaqMan Gene Expression Assays (Mm01221882_m1, Mm00477274_g1, Mm00479862_g1, Mm00478664_m1) from Applied Biosystems using the TaqMan 7900 Real-Time PCR system (Applied Biosystems). Raw data were normalized to Gapdh (Mm99999915) as a housekeeping gene. Relative expression quantification was achieved with the comparative ΔΔCt method.

### 4.5. Western Blot

Western blotting was performed using protein lysates from brain homogenates of APP/PS1 mice and APC cell lysates. The total protein was determined by detergent compatible (DC) Protein assay (Bio-Rad). For each lane, 50 μg of protein was subjected to sodium dodecyl sulfate polyacrylamide gel electrophoresis in a 12.5% or 10% gel and transferred to poly(vinylidene fluoride) (PVDF) membranes by tank blotting. Membranes were blocked with 5% bovine serum albumin, BSA (Carl Roth, Karlsruhe, Germany) in Tris-buffered saline (TBS) with Tween 20, TBST (0.05% Tween 20 in PBS or TBS, with and pH 7.4) for 1.5 h. Antibodies against BACE1 (dilution 1:500, Fisher Scientific GmbH, Schwerte, Germany), neprilysin (dilution, 1:2500, abcam, Cambridge, UK), AChE (1:500, Chemicon, Temecula, CA, USA), ionized calcium binding adaptor molecule 1, Iba-1 (dilution 1:1000, Wako, Neuss, Germany), ChAT (dilution 1:1000, Millipore, Burlington, MA, USA), Nestin (dilution 1:1000, BD Biosciences, San Jose, CA, USA), Sox 2 (1.1000, Santa Cruz), GS (dilution 1:1000, abcam), NeuroD1 (1:500, Gene Tex Inc, Irvine, CA, USA), EPO (1:500, Santa Cruz Biotechnology, Santa Cruz, CA, USA) and GAPDH (1:1000, loading control, Millipore) were used. For visualization of antibody binding, membranes were incubated with alkaline phosphatase- or Cy2/Cy5-conjugated antibodies for 2 h at room temperature (RT). Protein bands were visualized using chemiluminescence or fluorescence detection systems (Bio-Rad, Hercules, CA, USA). For imaging and densitometric analyses, a VersaDocTM 4000 MP imaging system (Bio-Rad) was used. Data were normalized to the respective densitometric values of loading controls (GAPDH).

### 4.6. Immunofluorescent Stainings

Horizontal cryosections (10 mm in thickness) from the brains of APP/PS1 mice and coverslips with WT and 3xTg-AD APC were fixed with methanol at −20 °C, washed, and incubated with antibodies against human Aβ (6E10 antibody, dilution 1:100; Signet, Dedham, MA, USA), glial fibrillary acidic protein GFAP (dilution 1:400; DacoCytomation, Glostrup, Denmark), acetylcholine (1:500, abcam), acetylcholine esterase (1:500, Chemicon), β tubulin III (clone TUJ1, 1:200, R&D Systems, Minneapolis, MN, USA), neprilysin (1:100, abcam), and choline acetyltransferase (1:500, Fisher Scientific GmbH) for 2 h at room temperature (RT). Sections were washed with PBS and further incubated with the respective Cy3- or fluorescein isothiocyanate (FITC)-conjugated antibodies (both from Dianova, Hamburg, Germany) for 1 h at RT. Thereafter, tissue slices and APCs were washed with PBS containing 0.05% Triton X-100 (Sigma, Steinheim, Germany) and coated with Vectashield mounting medium (Vector Laboratories, Burlingame, CA, USA) containing 4’,6 diamidino-2-phenylindole (DAPI, Linaris, Wertheim-Bettingen, Germany) and evaluated by fluorescence microscopy, using Olympus BX51 Microscope (Olympus Optical Co. Europe, Hamburg, Germany). Images were acquired by the digital camera F-View II and processed by the software Analysis DOKU^®^ (Soft Imaging System GmbH, Leinfelden-Echterdingen, Germany). The counts of neprilysin- and synaptophysin- positive cells were performed from three different experiments with *n* = 3 coverslips quantified in each.

### 4.7. Aβ42 ELISA and Multiplex Analysis of Cytokines

Aβ 42 in brain homogenates was quantified using the Aβ 42 Brain Elisa Kit (Millipore, Darmstadt, Germany). Reagent blank, standards (range 16–500 pg/mL), and samples were measured according to the manufacturer’s manual. Absorbances of the standards were plotted against their concentrations and unknowns are determined with a linear regression model. The result is presented as pg Aβ /mg total protein.

The measurement of interleukin-a (IL-1a), IL-1b, IL-4, IL-5, IL-6, IL-10, IL-12, tumor necrosis factor α (TNFα), interferon γ (IFNγ), and granulocyte-macrophage colony stimulating factor (GM-CSF) in the brain homogenates was performed with a mouse Procarta Plex multiplex immunoassay Kit (Thermo Fisher Scientific, Carlsbad, CA, USA) and a Luminex-100 system (Luminex Corporation, Austin, TX, USA) according to the manufacturer’s instructions.

### 4.8. Glutamine Synthetase Activity

GS activity in brain homogenates of APP/PS1 mice was measured by the colorimetric method, as described elsewhere [20,51]. The reaction of glutamine plus hydroxylamine to gamma glutamylhydroxamate catalyzed by GS was started by adding 50 µL of reaction mixture (50 mM imidazole/HCl-buffer (Merck, Darmstadt, Germany), pH 7.2, 2 mM MnCl2, 25 mM sodium arsenate, 0.16 mM adenosine diphosphate, ADP, 50 mM l-glutamine and 25 mM NH_2_×HCl (Sigma-Aldrich, Steinheim, Germany)) to the cell lysates. The reaction was stopped after 2 h incubation at 37 °C by 200 µL stop-solution consisting of 0.37 M FeCl_3_, 0.67 M HCl, 0.2 M trichlor acetic acid (Sigma-Aldrich, Steinheim, Germany). After 5 min centrifugation of the reaction mixture at 15,000× *g*, the supernatants were transferred to a 96-well plate and measured at 540 nm in a plate-reader. An external standard curve in the range between 0 to 5 mM l-glutamic acid gamma monohydroxamate (Sigma-Aldrich, Steinheim, Germany) was generated with 10 different concentration points.

### 4.9. Statistical Analyses

Normal distribution of all data sets was confirmed by Shapiro–Wilk test. For behavioral data, two way ANOVA with Holm Sidak’s multiple comparisons test was used to compare the data from different time points within one treatment group as well as the data sets from vehicle vs. losartan treated groups at each time point of the T-maze assessment (day 1 to 5). For comparison of two groups (control vs. losartan) in all other data sets t-test was employed. All statistical tests were two-tailed and statistical significance level was defined as 5%.

## Data Availability

Data can be made available by the corresponding author upon reasonable request.

## Supplementary Materials

The following are available online at https://www.mdpi.com/1424-8247/14/2/166/s1, Figure S1: Astroglial primary cultures (APC) from 3xTg-AD mouse brains were tracked without (a) and with 1μM losartan (b) for 24 h.
