# Supplementary material for: Losartan Improves Memory, Neurogenesis and Cell Motility in Transgenic Alzheimer’s Mice
Source: Pharmaceuticals (Basel). 2021 Feb 20;14(2):166. doi: 10.3390/ph14020166 (PMC7923419; doi:10.3390/ph14020166)
Supplement: Supplementary file 1 [file pharmaceuticals-14-00166-s001.pptx]

## Slide 1
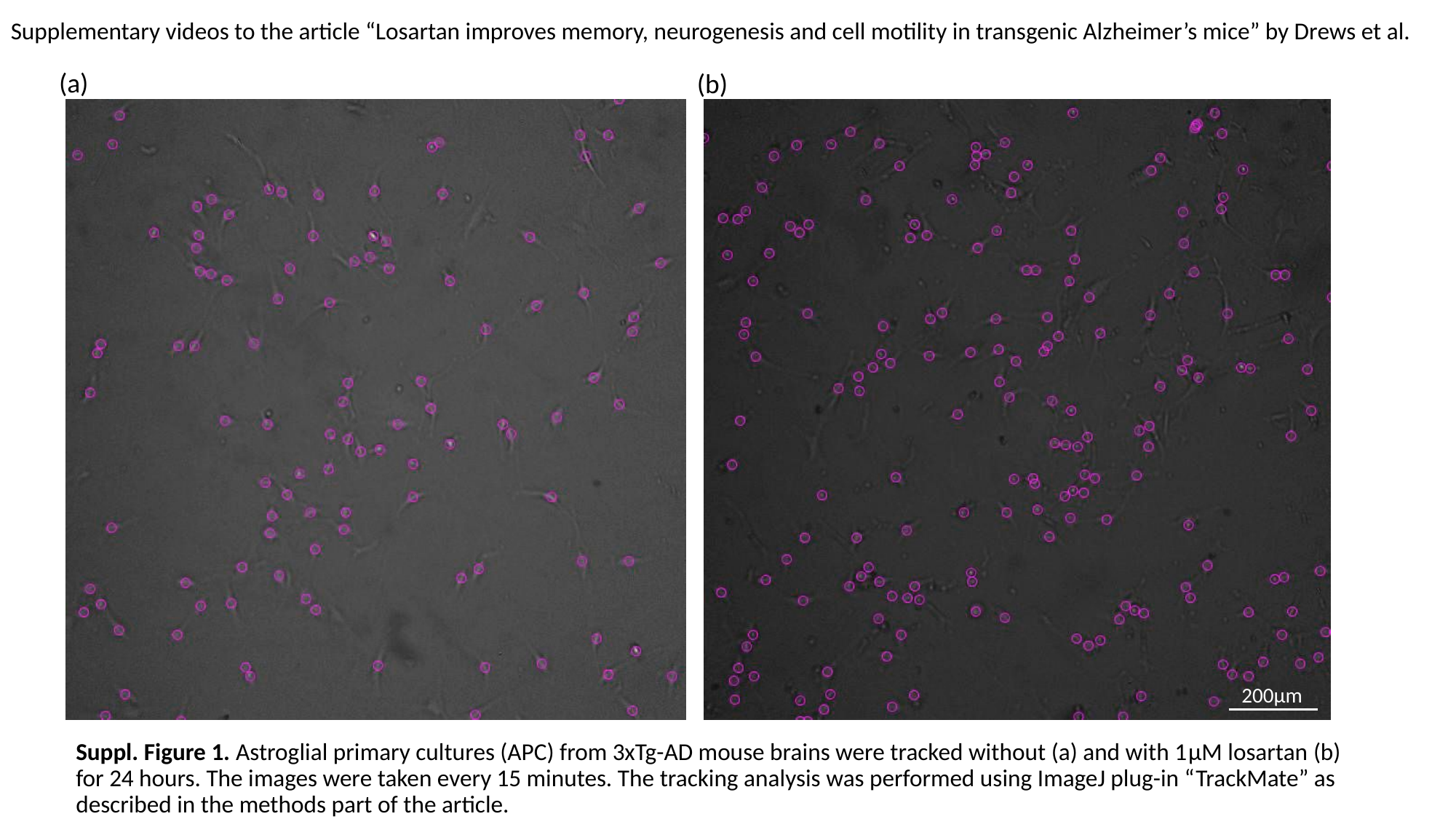

Supplementary videos to the article “Losartan improves memory, neurogenesis and cell motility in transgenic Alzheimer’s mice” by Drews et al.
(a)
(b)
200μm
Suppl. Figure 1. Astroglial primary cultures (APC) from 3xTg-AD mouse brains were tracked without (a) and with 1μM losartan (b) for 24 hours. The images were taken every 15 minutes. The tracking analysis was performed using ImageJ plug-in “TrackMate” as described in the methods part of the article.
